# Rates of glycaemic deterioration in a real-world population with type 2 diabetes

**DOI:** 10.1007/s00125-017-4519-5

**Published:** 2017-12-19

**Authors:** Louise A. Donnelly, Kaixin Zhou, Alex S. F. Doney, Chris Jennison, Paul W. Franks, Ewan R. Pearson

**Affiliations:** 1 Division of Molecular and Clinical Medicine, School of Medicine, University of Dundee, Dundee, DD1 9SY UK Scotland; 2 Department of Mathematical Sciences, University of Bath, Bath, UK; 3 Department of Clinical Science, Genetic and Molecular Epidemiology Unit, Lund University, Malmö, Sweden; 4 Department of Nutrition, Harvard School of Public Health, Boston, MA USA; 5 Department of Public Health and Clinical Medicine, Umeå University, Umeå, Sweden

**Keywords:** Coefficient of failure, Elderly, Electronic medical records, Glycaemic deterioration, Observational, Type 2 diabetes

## Abstract

**Aims/hypothesis:**

There is considerable variability in how diabetes progresses after diagnosis. Progression modelling has largely focused on ‘time to failure’ methods, yet determining a ‘coefficient of failure’ has many advantages. We derived a rate of glycaemic deterioration in type 2 diabetes, using a large real-world cohort, and aimed to investigate the clinical, biochemical, pharmacological and immunological variables associated with fast and slow rates of glycaemic deterioration.

**Methods:**

An observational cohort study was performed using the electronic medical records from participants in the Genetics of Diabetes Audit and Research in Tayside Study (GoDARTS). A model was derived based on an individual’s observed HbA_1c_ measures from the first eligible HbA_1c_ after the diagnosis of diabetes through to the study end (defined as insulin initiation, death, leaving the area or end of follow-up). Each HbA_1c_ measure was time-dependently adjusted for the effects of non-insulin glucose-lowering drugs, changes in BMI and corticosteroid use. GAD antibody (GADA) positivity was defined as GAD titres above the 97.5th centile of the population distribution.

**Results:**

The mean (95% CI) glycaemic deterioration for type 2 diabetes and GADA-positive individuals was 1.4 (1.3, 1.4) and 2.8 (2.4, 3.3) mmol/mol HbA_1c_ per year, respectively. A younger age of diagnosis, lower HDL-cholesterol concentration, higher BMI and earlier calendar year of diabetes diagnosis were independently associated with higher rates of glycaemic deterioration in individuals with type 2 diabetes. The rate of deterioration in those diagnosed at over 70 years of age was very low, with 66% having a rate of deterioration of less than 1.1 mmol/mol HbA_1c_ per year, and only 1.5% progressing more rapidly than 4.4 mmol/mol HbA_1c_ per year.

**Conclusions/interpretation:**

We have developed a novel approach for modelling the progression of diabetes in observational data across multiple drug combinations. This approach highlights how glycaemic deterioration in those diagnosed at over 70 years of age is minimal, supporting a stratified approach to diabetes management.

**Electronic supplementary material:**

The online version of this article (10.1007/s00125-017-4519-5) contains peer-reviewed but unedited supplementary material, which is available to authorised users.


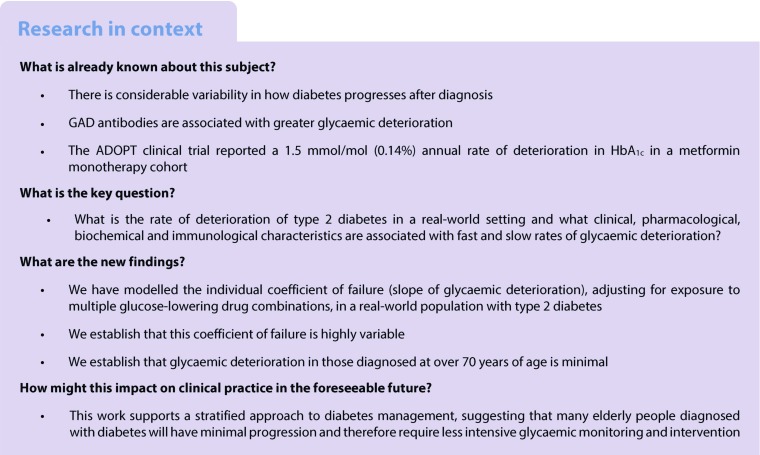


## Introduction

Type 2 diabetes is a progressive disease, primarily characterised by beta cell failure [1, 2]. This progression is manifested clinically by a deterioration in HbA_1c_ levels over time, despite lifestyle and increased pharmacological interventions. However, the rate at which diabetes progresses is highly variable between individuals. Some individuals have a rapid deterioration and advance to insulin therapy quickly, whereas others can be adequately treated with non-insulin glucose-lowering medication for in excess of 20 years. Gaining insight into why some individuals progress rapidly while others do not will enable a more stratified approach to the management of type 2 diabetes by identifying subgroups who may require different management depending on their likelihood of diabetes progression.

Previous studies have investigated factors associated with the rate of diabetes progression. However, these studies have only reported an outcome based on progression to glucose-lowering medications (i.e. time to initiation of non-insulin glucose-lowering medication, failure of monotherapy or time to insulin therapy) [1, 3–9]. In these studies, younger age at diagnosis and insufficient beta cell function were consistently associated with faster progression of diabetes. The UK Prospective Diabetes Study (UKPDS) reported that the presence of positive GAD antibody (GADA) concentrations predicted an increased likelihood of requirement for insulin [3]. Other less well established associations were female sex, low BMI (defined as <30 kg/m^2^), weight gain, lower HDL-cholesterol and higher serum creatinine. In addition, we have previously reported that risk of progression, defined by a requirement for insulin treatment, is associated with normal weight or obesity (a U-shaped relationship), and higher triacylglycerol and lower HDL-cholesterol levels [6].

The studies outlined rely on defining an endpoint, such as a glycaemic threshold or starting a new drug. These ‘time to failure’ approaches are problematic, particularly in the real world, where decisions to start a drug may be subject to prescriber or patient inertia, or where fluctuations in HbA_1c_, for example due to lifestyle change resulting from life or health status events, can trigger a failure event. A ‘coefficient of failure’ measure has been proposed to avoid these difficulties—in essence, deriving a rate of glycaemic deterioration for each individual [10]. This approach was applied to the UKPDS study, which reported a coefficient of failure of 3.7 mmol/mol (0.34%) per year with chlorpropamide treatment [10], and to the A Diabetes Outcome Progression Trial (ADOPT) study, which described a rate of glycaemic deterioration of 1.5 mmol/mol (0.14%) HbA_1c_ per year in the metformin monotherapy arm [11]. However, to our knowledge, no studies have been reported describing the coefficient of failure in settings outside these clinical trials of monotherapy. Determining rates of deterioration in a population over time is challenging as underlying disease severity reflects not only observed HbA_1c_, but also lifestyle and pharmacological interventions.

The aim of this study was to derive a model for the rate of deterioration of type 2 diabetes (coefficient of failure) in a large population-based cohort and to investigate the clinical, pharmacological, biochemical and immunological characteristics associated with fast and slow rates of glycaemic deterioration.

## Methods

An observational cohort study was performed using comprehensive electronic medical records from individuals in the Genetics of Diabetes Audit and Research in Tayside Study (GoDARTS) database, which has previously been described elsewhere [12, 13]. In short, this contains detailed information on all encashed prescriptions from 1994 onwards in Tayside, Scotland, as well as all biochemistry and BMI measures. Therefore, for each individual we have a comprehensive longitudinal record of diabetes therapy and glycaemic control.

The GoDARTS study was approved by the Tayside Committee on Medical Research Ethics, and informed consent was obtained from all participants (REC reference 053/04). The GoDARTS cohort and the research question outlined here were studied as part of the Diabetes Research on Patient Stratification (DIRECT) study, an EU Seventh Framework Programme (FP7) Innovative Medicines Initiative (see www.direct-diabetes.org) project.

### Study population

Diagnosis of diabetes was defined as the date of the first HbA_1c_ measurement ≥48 mmol/mol (6.5%) (based on the recommended cut-off point for diagnosing diabetes) or the first prescription of glucose-lowering medication, following a clinical diagnosis of type 2 diabetes. Individuals were followed from diagnosis until insulin initiation, death, leaving the area or end of follow-up (30 September 2015), whichever came first. To ensure sufficient prescribing information and longitudinal HbA_1c_ and BMI measurements, individuals had to have been diagnosed with diabetes on or after 1 January 1994 to be eligible for the study.

### GADA

GADA were measured at the time of recruitment into GoDARTS, allowing us to define a subgroup of individuals who were ‘GADA positive’ (defined as ≥11 U/l [97.5th centile]), whom we would expect to have a more rapid progression of diabetes and show different clinical covariates associated with progression compared with individuals with type 2 diabetes [3].

### Study criteria

The underlying assumption of our progression model was that change in HbA_1c_ over time was linear, and this was supported by the Belfast Diet Study, which reported two linear phases before and after the diagnosis of diabetes [1]. Some individuals who had a high HbA_1c_ at diagnosis and subsequent marked improvement in HbA_1c_ did not fulfil this assumption of linearity. Therefore, for all individuals, we restricted the starting HbA_1c_ value to an upper limit of 64 mmol/mol (8%), and allowed 1 year from diagnosis to reach this target HbA_1c_ level.

The first HbA_1c_ measure satisfying the inclusion criteria was defined as the study start for that individual. At least two subsequent HbA_1c_ measurements were required for an individual to be included in the analysis. In addition, individuals were required to have a BMI measurement at diagnosis (defined as the average of all available measures ±1 year from the diagnosis of diabetes) and at least two subsequent BMI measures during the follow-up period. A small number of individuals were also excluded during the analysis as they had fewer than three HbA_1c_ and/or BMI measures after outlying data points had been removed (see below).

### Outcome

A model was derived for each individual’s glycaemic deterioration rate based on observed HbA_1c_ measures from the first eligible HbA_1c_ through to study end. HbA_1c_ measures were adjusted time-dependently for the following measures:

1. Non-insulin glucose-lowering drugs. Untreated measures were the reference group, defined as measures prior to initiation of glucose-lowering drugs. As metformin was the most commonly prescribed glucose-lowering drug and we expected to observe a dose-dependent relationship with HbA_1c_ [14], we divided daily dose into three groups (<1 g, 1 to <2 g, and ≥2 g). The other glucose-lowering drugs were grouped solely by drug class, either because there was no evidence of a dose-dependent relationship with HbA_1c_ or because the limited number of measures would result in multiple, small groups. Glucose-lowering drugs were further grouped into monotherapy, and combinations of dual and triple therapy.
2. BMI change. This was expressed as the percentage change from BMI at diagnosis and categorised into three groups: stable weight (defined as no more than 5% change), significant weight gain (increase of ≥5%), and significant weight loss (decrease of ≥5%).
3. Glucocorticoid use. A widely recognised side effect of glucocorticoids is to temporarily raise HbA_1c_ [15], and a significant proportion of individuals were prescribed glucocorticoids during the study period. We categorised use as ‘yes’ or ‘no’ at each HbA_1c_ measure.

### Covariates

The following covariates were included in the model: age at diabetes diagnosis, sex, calendar year of diagnosis and a variable indicating high baseline HbA_1c_ at diagnosis (i.e. initial HbA_1c_ >64 mmol/mol [8%]). BMI, HDL-cholesterol and triacylglycerols were also included, defined as the average of all measures ±1 year from diagnosis.

### Statistical analysis

A linear mixed effects model was fitted. As the time intervals between HbA_1c_ measurements were more or less unique to each individual, the ‘continuous time/continuous space’ spatial data covariance structure provided within the PROC MIXED procedure in SAS 9.4 (SAS Institute, Cary, NC, USA) was used to describe the covariance structure among the errors.

We began by fitting a model with both a fixed and random intercept and slope, and adjustment for non-insulin glucose-lowering drugs, glucocorticoid use and changes in BMI over time, fitted as fixed effects. The Studentised residuals were examined and any HbA_1c_ measures >3 SD from the mean were removed as these values were considered likely to be outliers for that individual.

We then ran the model again for type 2 diabetes and GADA-positive individuals separately and compared the individual rates of glycaemic deterioration. These were calculated by adding together each individual’s random slope with the population average (fixed) slope.

The model was then expanded in individuals with type 2 diabetes only, owing to small numbers in the GADA-positive group, to include the baseline clinical covariates of interest. To model the effect of each covariate on glycaemic deterioration, an interaction term between the covariate and time was included. We fitted univariate models in which baseline covariates were added singly, and a multivariate model that included all univariately significant covariates together. Age at diagnosis was split into four age bands (<50, 50–<60, 60–<70 and ≥70 years), and BMI was split into five categories based on WHO definitions (<25, 25–<30, 30–<35, 35–<40 and ≥40 kg/m^2^). HDL-cholesterol and triacylglycerol concentrations were split into four clinically meaningful bands (HDL-cholesterol: <1, 1–<1.2, 1.2–<1.4 and ≥1.4 mmol/l; triacylglycerols: <1.5, 1.5–<2.5, 2.5–<3.5 and ≥3.5 mmol/l), with an additional ‘missing’ group created to avoid excluding individuals with missing values from the multivariate model. Calendar year of diagnosis was divided into quartiles.

All analyses were performed using SAS, and *p* < 0.05 was considered statistically significant in all analyses.

## Results

### Individual characteristics

From a total of 6728 individuals with type 2 diabetes, 5491 (82%) met the study inclusion criteria. A detailed flow chart of the study population derivation is presented in ESM Fig. 1. The median (with interquartile range [IQR]) study follow-up time was 9.4 (6.1–12.4) years, and the median (IQR) numbers of HbA_1c_ and BMI measures per individual were 21 (14–29) and 20 (13–29), respectively. A total of 121,972 HbA_1c_ measures were generated for the 5491 individuals.

A comparison of characteristics of individuals included in and excluded from the study is presented in Table 1. Individuals not meeting the study criteria were younger and had lower HDL-cholesterol, higher triacylglycerol and higher HbA_1c_ measurements at diagnosis. In addition, there were higher proportions of GADA-positive individuals and/or participants who had progressed to insulin therapy by the end of the study period. The characteristics of the three subgroups within the study population are also presented in Table 1. As expected, GADA-positive individuals were diagnosed at a younger age and with a lower BMI, lower triacylglycerols and higher HDL-cholesterol, and were more likely to progress to insulin than were individuals with type 2 diabetes.

**Table 1:** Characteristics at diagnosis of individuals in the study by subgroups

Variable	All individuals					Study population			
						GADA-positive	Type 2 diabetes		
	Included	n	Excluded	n	*p* value		HbA_1c_ at diagnosis met study criteria	HbA_1c_ >64 mmol/mol, 8%, at diagnosis	*p* value
*N*	5491		1237			149	3574	1768	
Age, years	61.5 ± 11.1	5491	58.4 ± 12.1	1237	<0.0001	59.5 ± 12.3	62.2 ± 11.0	60.3 ± 11.0	<0.0001
Male, *n* (%)	3086 (56.2)	5491	653 (52.8)	1237	0.0291	73 (49.0)	1974 (55.2)	1039 (58.8)	0.0142
BMI, kg/m^2^	31.4 ± 5.9	5491	31.4 ± 5.9	986	0.6912	29.3 ± 5.7	31.6 ± 6.0	31.2 ± 5.8	0.0141
HDL-cholesterol, mmol/l	1.21 ± 0.32	5227	1.18 ± 0.32	1000	0.0120	1.25 ± 0.30	1.22 ± 0.32	1.17 ± 0.30	<0.0001
Triacylglycerol, mmol/l	2.3 (1.6–3.2)	3960	2.5 (1.7–3.8)	747	0.0008	2.0 (1.3–2.6)	2.2 (1.6–3.2)	2.4 (1.7–3.5)	<0.0001
HbA_1c_ at diagnosis									
mmol/mol	64.5 ± 20.0	5491	82.4 ± 24.8	1178	<0.0001	70.3 ± 23.4	52.8 ± 5.4	87.4 ± 18.3	<0.0001
%	8.0 ± 1.8	5491	9.7 ± 2.3	1178		8.6 ± 2.2	6.9 ± 0.5	10.1 ± 1.7	
HbA_1c_ at inclusion									
mmol/mol	53.4 ± 6.1	5491		1178		53.6 ± 5.6	52.8 ± 5.4	54.5 ± 7.1	<0.0001
%	7.0 ± 0.6	5491		1178		7.1 ± 0.5	6.9 ± 0.5	7.1 ± 0.6	
GADA-positive, *n* (%)	149 (2.7)	5491	99 (8.0)	1237	<0.0001	–	–	–	–
Progressed to insulin by study end, *n* (%)	1145 (20.9)	5491	649 (52.5)	1237	<0.0001	67 (45.0)	576 (16.1)	502 (28.4)	<0.0001

Data are mean (SD), *n* (%) or median (IQR)

Comparison was by *t* test for continuous variables (triacylglycerols were log_10_-transformed) and χ^2^ test for categorical variables

### Linear mixed model-derived effects

The linear mixed model included 76 different drug combinations as fixed effects. These represent the model-derived estimates for HbA_1c_ reduction by a particular drug combination compared with no treatment. The drug effects for the most commonly prescribed combinations (defined as >500 HbA_1c_ measures) are presented in ESM Table 1. There was a total of 33,243 (27.2%) untreated measures from 3736 (68%) individuals. We observed a dose-dependent relationship with metformin with <1 g, 1 to <2 g and ≥2 g per day lowering HbA_1c_ on average (95% CI) by 0.8 (0.4, 1.3) mmol/mol (0.08% [0.03%, 0.12%]), 2.8 (2.5, 3.0) mmol/mol (0.25% [0.23%, 0.28%]) and 4.2 (3.9, 4.6) mmol/mol (0.39% [0.36%, 0.42%]), respectively. A >5% BMI increase was associated with an average (95% CI) HbA_1c_ increase of 1.2 (1.0, 1.3) mmol/mol (0.11% [0.09%, 0.12%]). Conversely, a >5% reduction in BMI was associated with a decrease in HbA_1c_ of on average (95% CI) 2.0 (1.9, 2.2) mmol/mol (0.19% [0.17%, 0.20%]). A total of 4958 (4%) of HbA_1c_ measures were taken while the participant was on glucocorticoids; these were associated with an average (95% CI) HbA_1c_ increase of 3.2 (2.8, 3.5) mmol/mol (0.29% [0.26%, 0.32%]) (BMI and glucocorticoid data not shown).

### Rates of glycaemic deterioration in type 2 diabetic and GADA-positive individuals

**Fig. 1:**
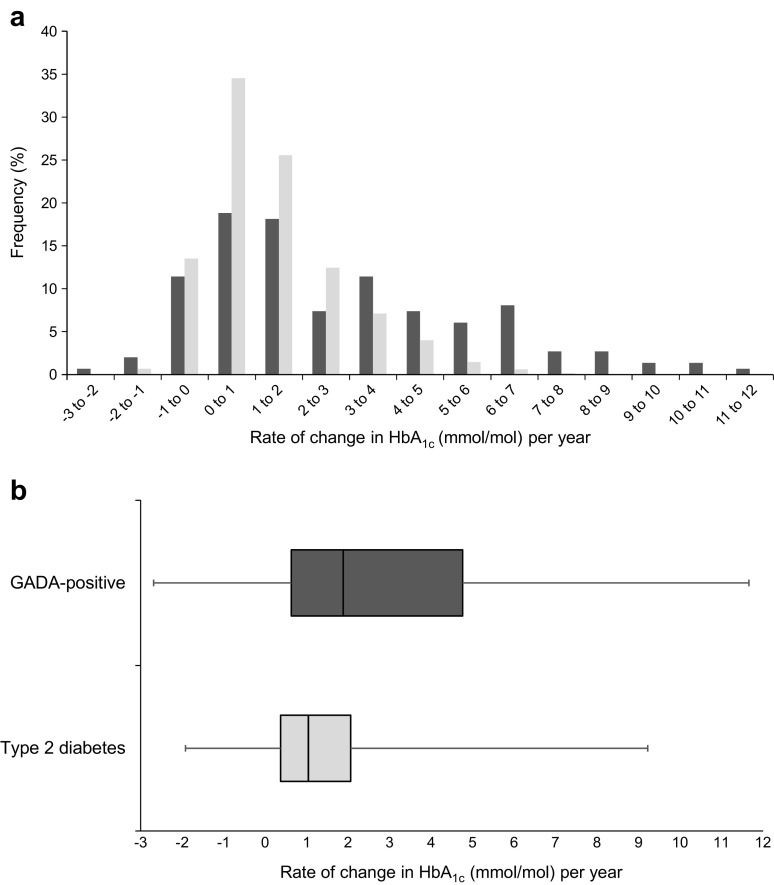
Distribution of rate of glycaemic deterioration (increase in adjusted HbA_1c_ per year characterised in mmol/mol units), presented as a histogram (**a**) and box-and-whisker plot (**b**). Light grey, type 2 diabetes; dark grey, GADA positivity. Ranges in (**a**) are from −3 to <−2; −2 to <−1 etc.

The model-derived individual glycaemic deterioration rate was the rate of change of HbA_1c_ per year after adjusting for the effect of drug treatment and change in BMI. The distribution of the individuals’ glycaemic deterioration rate is presented in Fig. 1, with type 2 diabetic (*n* = 5342) and GADA-positive (*n* = 149) individuals presented separately. The mean (95% CI) coefficient of failure for individuals with type 2 diabetes was 1.4 (1.3, 1.4) mmol/mol (0.12% [0.12%, 0.13%]) per year, and the median (IQR) was 1.0 (0.4–2.1) mmol/mol (0.09% [0.03–0.10%]). By comparison, the coefficient of failure (95% CI) for GADA-positive individuals was reached approximately twice as rapidly, at 2.8 (2.4, 3.3) mmol/mol (0.25% [0.20%, 0.31%]) per year with a median (IQR) 1.9 (0.6–4.8) mmol/mol (0.17% [0.06–0.44%]) (*p* < 0.0001).

### Clinical characteristics associated with glycaemic deterioration in type 2 diabetes

To investigate which clinical covariates other than GADA positivity were associated with glycaemic deterioration, we expanded the model to include baseline clinical covariates within the group with type 2 diabetes. The results for the overall model are presented in Table 2.

**Table 2:** Differences in estimated glycaemic deterioration rates in individuals with type 2 diabetes

Variable	n	Univariate analysis		Multivariate analysis	
		Unadjusted coefficient (95% CI)^a^	*p* value	Adjusted coefficient (95% CI)^b^	*p* value
Age, years					
<50	823	1.80 (1.63, 1.97)	<0.0001	1.67 (1.49, 1.85)	<0.0001
50–<60	1430	0.96 (0.81, 1.11)	<0.0001	0.89 (0.74, 1.04)	<0.0001
60–<70	1820	0.42 (0.28, 0.57)	<0.0001	0.38 (0.24, 0.52)	<0.0001
≥70	1269	REF		REF	
Sex					
Male	3013	0.14 (0.03, 0.25)	0.0107	0.06 (−0.04, 0.17)	0.2370
Female	2329	REF		REF	
Year diagnosed					
<2001	1567	1.50 (1.33, 1.67)	<0.0001	1.55 (1.39, 1.72)	<0.0001
2001–<2003	1318	0.36 (0.26, 0.45)	<0.0001	0.38 (0.28, 0.48)	<0.0001
2003–<2006	1263	0.10 (0.03, 0.16)	0.0021	0.10 (0.04, 0.17)	0.0010
≥2006	1194	REF		REF	
Baseline HbA_1c_ >64 mmol/mol:					
No	3574	REF		REF	
Yes	1768	0.19 (0.08, 0.31)	0.0017	0.07 (−0.04, 0.18)	0.2300
BMI (kg/m^2^):					
<25	533	−0.08 (−0.28, 0.11)	0.4008	0.05 (−0.14, 0.23)	0.6387
25–<30	1890	REF		REF	
30–<35	1703	0.20 (0.07, 0.33)	0.0023	0.07 (−0.05, 0.20)	0.2371
35–<40	774	0.27 (0.10, 0.44)	0.0016	−0.02 (−0.19, 0.14)	0.7887
≥40	442	0.76 (0.55, 0.97)	<0.0001	0.26 (0.06, 0.47)	0.0128
HDL-cholesterol (mmol/l):					
<1	1275	0.60 (0.44, 0.76)	<0.0001	0.21 (0.05, 0.38)	0.0107
1–<1.2	1524	0.41 (0.25, 0.56)	<0.0001	0.18 (0.03, 0.34)	0.0188
1.2–<1.4	1168	0.15 (−0.01, 0.32)	0.0673	0.03 (−0.13, 0.19)	0.7291
≥1.4	1119	REF		REF	
Missing	256	0.01 (−0.25, 0.26)	0.9266	−0.17 (−0.42, 0.09)	0.1850
Triacylglycerol (mmol/l):					
<1.5	790	REF		REF	
1.5–<2.5	1391	0.08 (−0.10 to 0.26)	0.4173	−0.01 (−0.18, 0.17)	0.9315
2.5–<3.5	858	0.16 (−0.03, 0.36)	0.1110	−0.04 (−0.23, 0.15)	0.6677
≥3.5	819	0.36 (0.16, 0.56)	0.0005	−0.03 (−0.22, 0.17)	0.7767
Missing	1484	−0.03 (−0.21, 0. 51)	0.7477	0.07 (−0.11, 0.24)	0.4402

^a^Units are mmol/mol HbA_1c_ per year, adjusted only for glucose-lowering medication, steroid use and change in BMI

^b^Units are mmol/mol HbA_1c_ per year, adjusted for glucose-lowering medication, steroid use, change in BMI, age at diagnosis, sex, year diagnosed, baseline HbA_1c_ group, BMI, triacylglycerols and HDL-cholesterol

Values are expressed as the absolute difference in progression rate between the study group and the reference group. Positive values mean that the glycaemic deterioration rate is faster than the reference group

In the univariate analyses, younger age, male sex, HbA_1c_ >64 mmol/mol (8%) at presentation, earlier calendar year of diagnosis, higher BMI, lower HDL-cholesterol and higher triacylglycerols were all associated with a higher rate of glycaemic deterioration. In the multivariate model, younger age at diagnosis, lower HDL-cholesterol, higher BMI and earlier calendar year of diagnosis were independently associated with a higher rate of glycaemic deterioration: individuals diagnosed younger than 50 years of age deteriorated on average (95% CI) 1.67 (1.49, 1.85) mmol/mol (0.15% [0.14%, 0.17%]) HbA_1c_ per year faster than individuals diagnosed over 70 years of age; individuals with an HDL-cholesterol <1 mmol/l deteriorated on average (95% CI) 0.21 (0.05, 0.38) mmol/mol (0.02% [0.01%, 0.04%]) per year more quickly than individuals with an HDL-cholesterol ≥1.4 mmol/l; individuals with a BMI ≥40 kg/m^2^ deteriorated on average (95% CI) 0.26 (0.06, 0.47) mmol/mol (0.02% [0.01%, 0.04%]) per year faster than individuals with a BMI of 25–30 kg/m^2^; and individuals diagnosed prior to 2001 deteriorated on average (95% CI) 1.55 (1.39, 1.72) mmol/mol (0.14% [0.13%, 0.16%]) per year faster than individuals diagnosed in or after 2006.

**Fig. 2:**
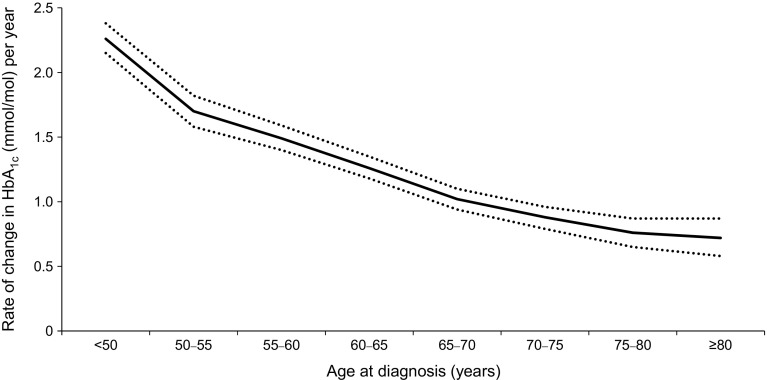
Mean (95% CI) rate of glycaemic deterioration (increase in adjusted HbA_1c_ per year characterised in mmol/mol units), by age at diagnosis. Ranges are 50–<55; 55–<60 etc.

To further investigate the relationship between younger age at diagnosis and higher rate of glycaemic deterioration, the mean (95% CI) coefficient of failure grouped by 5 year age bands for individuals with type 2 diabetes is presented in Fig. 2. Of the individuals diagnosed at under 50 years of age, 15% had a glycaemic deterioration rate of >4.4 mmol/mol (0.4%) per year, compared with 1.5% of the individuals diagnosed aged over 70 years. Conversely, 66% of the individuals diagnosed over 70 years old had a glycaemic deterioration rate <1.1 mmol/mol (0.1%) per year compared with just 24% of the individuals diagnosed under 50 years of age.

## Discussion

In this large, observational, population-based study with a maximum follow-up period of over 20 years, we have applied a novel approach to modelling the progression of diabetes. We have shown that, in a real-world setting, the underlying mean coefficient of failure (rate of glycaemic deterioration) in individuals with type 2 diabetes is 1.4 mmol/mol (0.12%) HbA_1c_ per year, and in GADA-positive individuals it is faster, with a mean rate of 2.8 mmol/mol (0.25%) per year. Furthermore, our results suggest that individuals with type 2 diabetes who deteriorate the fastest are those diagnosed under 50 years old, and that there is very limited deterioration in those diagnosed over the age of 70.

We report a coefficient of failure in individuals with type 2 diabetes comparable to that of the ADOPT clinical trial, which reported a 1.5 mmol/mol (0.14%) annual rate of deterioration in HbA_1c_ in a metformin monotherapy cohort [11]. Moreover, we know from the UKPDS that GADA positivity is a strong predictor of diabetes progression [3], and here we have shown that GADA-positive individuals progress approximately two times faster than individuals with type 2 diabetes. In the group of individuals who are not known to be GADA-positive, faster diabetes progression is associated with clinically small but statistically significant differences in BMI and HDL-cholesterol, in keeping with the insulin resistance phenotype.

Our findings are in accordance with other studies that have reported the association between younger age at diagnosis and faster progression of diabetes [1, 4–8]. Individuals diagnosed younger than 50 years of age progress rapidly compared with individuals diagnosed over the age of 70 (Fig. 2), and as HbA_1c_ at diagnosis is higher in the younger than the older group (mean [95% CI]: 66.4 [65.1, 67.8] vs 61.9 [60.8, 62.9] mmol/mol; 8.23% [8.11%, 8.35%] vs 7.81% [7.71%, 7.90%]; *p* < 0.0001), this suggests that individuals diagnosed younger may benefit from being treated more aggressively with earlier initiation of glucose-lowering medications, particularly if future therapies can be established to delay progression. The finding that, in the real world, 66% of individuals with type 2 diabetes diagnosed after the age of 70 years progress at a rate <1.1 mmol/mol (0.1%) per year, and that only 1.5% progress at a rate >4.4 mmol/mol (0.4%) per year, is striking and highlights how glycaemic monitoring and management in those diagnosed at over 70 years may not need to be as aggressive as those diagnosed under 50 years of age.

We have previously reported that earlier calendar year of diagnosis is associated with risk of progression, as defined by requirement for insulin treatment [6]. We believe that this reflects a change in practice over time, with possibly two factors influencing progression rate. First, individuals may be diagnosed earlier in more recent years due to screening or increased awareness. This is supported by the observation that individuals diagnosed prior to 2001 have a higher HbA_1c_ at diagnosis than those diagnosed in or after 2006 (mean [95% CI]: 65.1 [64.1, 66.0] vs 60.5 [59.5, 61.6] mmol/mol; 8.11% [8.02%, 8.20%] vs 7.68% [7.58%, 7.78%]; *p* < 0.0001). Second, with increasing calendar years, there may be improved general health and better treatment of all diabetes risk factors that may impact on rates of progression.

In this analysis, we included a group who at diagnosis had a high HbA_1c_ of >64 mmol/mol (8%) but whose HbA_1c_ level fell to meet the inclusion criteria within the first year. Many mechanisms may underlie this pattern, but one possible explanation is that these are a group who initially present with high HbA_1c_ driven by gluco-lipotoxcity, who subsequently show rapid improvement with dietary and drug treatment. It is interesting to note that, in the multivariate analysis, this group, despite an initial high HbA_1c_, subsequently progressed at the same rate as those whose initial HbA_1c_ was <64 mmol/mol (8%).

The aim of this study was to derive a ‘rate of deterioration’ or ‘coefficient of failure’, which we believe has many advantages over a time to failure model. However, a number of assumptions have been made in order to develop this model. First, we assume a linear deterioration in HbA_1c_; this is supported by the Belfast Diet Study, which reported two linear phases before and after the diagnosis of diabetes [1]. However, there may be individuals who do not follow this linear decline who are not well accounted for in our model. Second, individuals were excluded from entry into the model largely because they had a high HbA_1c_ at diagnosis that did not fall below 64 mmol/mol (8%) within the first year, or because they had too few HbA_1c_ measures before they progressed onto insulin. As such, our model excludes those with the most aggressive disease and/or those who present late with a high HbA_1c_, and focuses on those diagnosed close to onset of diabetes or with less aggressive disease. Therefore our coefficients of failure are likely to underestimate the true progression rate in the population. Third, we define diabetes diagnosis as a first HbA_1c_ ≥48 mmol/mol (6.5%) following a clinical diagnosis of type 2 diabetes, and as an individual may have a diagnostic glucose level but an HbA_1c_ <48 mmol/mol (6.5%), this means that we will underestimate the duration of diabetes and overestimate the slope in some individuals. Finally, the fact that we are studying real-world individuals in clinical practice means that we lack some key measures that may be important for glycaemic deterioration, such as measures of beta cell function and insulin resistance.

In summary, we have developed a novel approach to model the coefficient of failure in observational data across multiple drug combinations. This approach may be valuable in investigating biomarker or genomic determinants of diabetes progression in bioresources. In addition, although our current model derives a ‘global’ rate of deterioration from diagnosis to insulin initiation, future developments may allow investigation of how the rate varies for therapies for diabetes and for other conditions. We confirm that GADAs are associated with greater glycaemic deterioration, and for the first time quantify the rate of glycaemic deterioration in the elderly. Our findings of minimal glycaemic deterioration in this elderly-onset group has important implications for stratifying diabetes care, suggesting that less intensive glycaemic monitoring and management is required for this group.

## Electronic supplementary material

ESM (PDF 112 kb)

## Abbreviations

ADOPT: A Diabetes Outcome Progression Trial
DIRECT: Diabetes Research on Patient Stratification study
GADA: GAD antibody
GoDARTS: Genetics of Diabetes Audit and Research in Tayside Study
IQR: Interquartile range
UKPDS: UK Prospective Diabetes Study
