# Supplementary material for: Rates of glycaemic deterioration in a real-world population with type 2 diabetes
Source: Diabetologia. 2017 Dec 19;61(3):607–15. doi: 10.1007/s00125-017-4519-5 (PMC6448965; doi:10.1007/s00125-017-4519-5)

ESM table 1: Drug effects for most commonly prescribed treatment(s) derived from linear mixed model

Drug combination	Estimate (95% CI)	N (total number of measures)
Untreated	REF	33,243 (27.2%)
MONOTHERAPY:		
Metformin:<br><1g<br>≥1g - <2g<br>≥2g	 -0.8 (-1.3, -0.4)<br>-2.8 (-3.0, -2.5)<br>-4.2 (-4.6, -3.9)	 1961(1.6%)<br>21317(17.5%)<br>10288(8.4%)
SU	-3.4 (-3.7, -3.2)	15781(12.9%)
TZD	-7.4 (-8.3, -6.5)	695(0.6%)
DUAL THERAPY:		
Metformin + SU:<br><1g<br>≥1g - <2g<br>≥2g	 -4.5 (-5.2, -3.8)<br>-6.7 (-7.0, -6.3)<br>-9.2 (-9.5, -8.8)	 781(0.6%)<br>8729(7.2%)<br>11344(9.3%)
Metformin + TZD:<br>≥1g - <2g<br>≥2g	 -8.2 (-9.0, -7.5)<br>-8.9 (-9.5, -8.4)	 1190(1.0%)<br>2505(2.1%)
SU + TZD	-9.9 (-10.5, -9.3)	2045(1.7%)
≥2g Metformin + DPP4	-7.0 (-7.8, -6.2)	1099(0.9%)
TRIPLE THERAPY:		
Metformin + SU + TZD:<br>≥1g - <2g<br>≥2g	 -13.7 (-14.4, -12.9)<br>-16.5 (-17.1, -16.0)	 935(0.8%)<br>3063(2.5%)
Metformin + SU + DPP4:<br>≥1g - <2g<br>≥2g	 -11.6 (-12.6, -10.7)<br>-14.3 (-14.9, -13.7)	 593(0.5%)<br>2155(1.8%)
≥2g *Metformin + SU + GLP1:*	-17.3 (-18.3, -16.3)	699(0.6%)

SU: Sulphonylureas; TZD: Thiazolidinediones; DPP4: Dipeptidyl Peptidase-4 Inhibitors. GLP1: glucagon-like peptide agonists. The coefficient presented in the table is the effect of the drug(s) on HbA_{1c} *characterised in mmol/mol units, with untreated measures as the reference group.*

ESM Fig. 1: Study population derivation

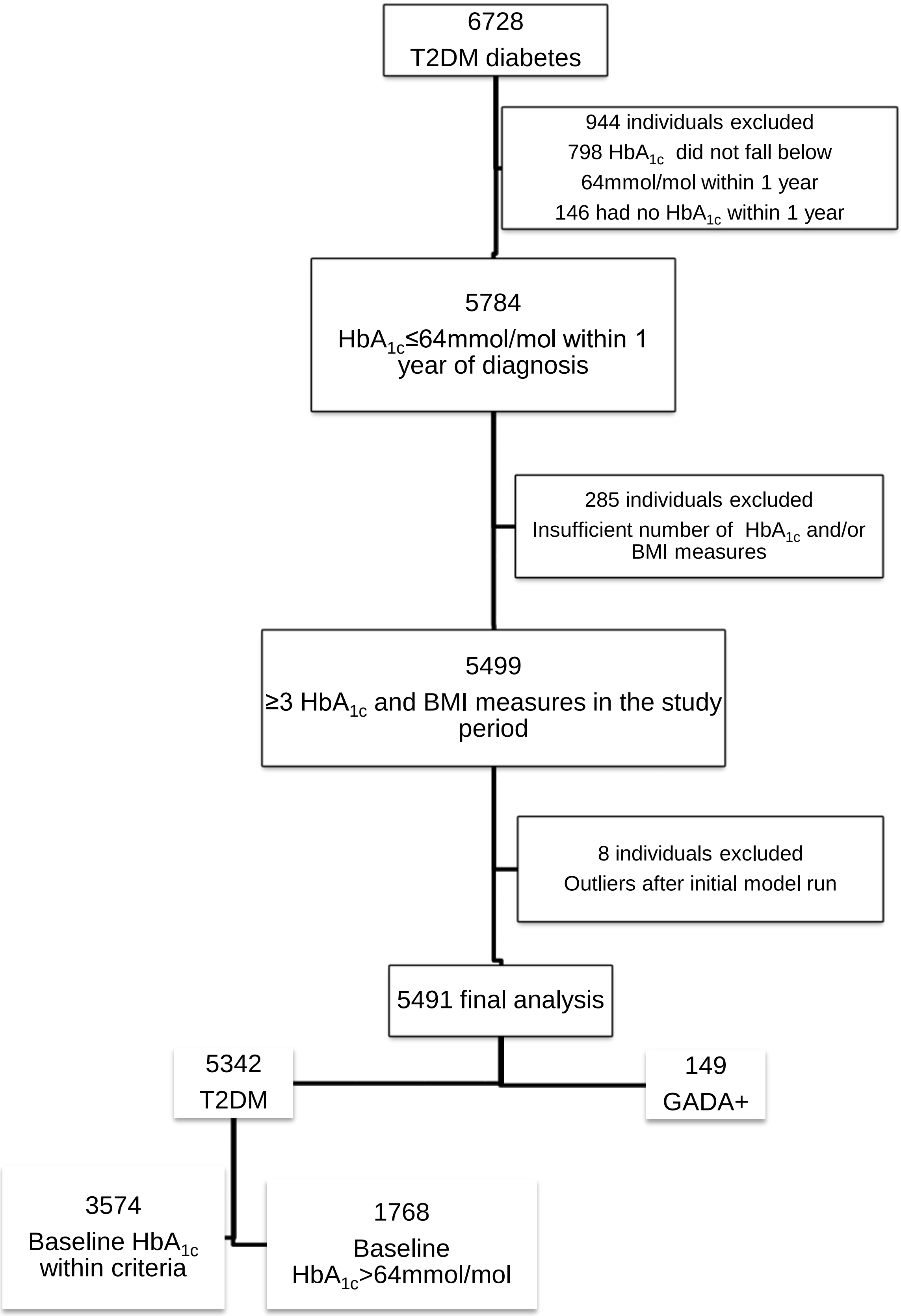

Supplement: Supplementary file 1 — (PDF 112 kb) [file 125_2017_4519_MOESM1_ESM.pdf]
